# The Combined Effect of Multisensory Stimulation and Therapist Support on Physical and Mental Health of Older Adults Living in Nursing Homes: Pilot Randomized Controlled Trial

**DOI:** 10.2196/55042

**Published:** 2025-01-14

**Authors:** Sewar Khatib, Yuval Palgi, Yoni K Ashar, Natalya Polyvyannaya, Pavel Goldstein

**Affiliations:** 1 School of Public Health University of Haifa Haifa Israel; 2 Department of Gerontology University of Haifa Haifa Israel; 3 Faculty of Medicine University of Colorado Anschutz Medical Campus Aurora, CO United States; 4 Department of General and Applied Psychology Kazakh National University Almaty Kazakhstan; 5 Faculty of Business, Media and Management International Informational Technologies University Almaty Kazakhstan

**Keywords:** Snoezelen room, mental health, sensory stimulation environment, social support, nursing homes, older adults

## Abstract

**Background:**

Increasing life expectancy has led to a rise in nursing home admissions, a context in which older adults often experience chronic physical and mental health conditions, chronic pain, and reduced well-being. Nonpharmacological approaches are especially important for managing older adults’ chronic pain, mental health conditions (such as anxiety and depression), and overall well-being, including sensory stimulation (SS) and therapist support (TS). However, the combined effects of SS and TS have not been investigated.

**Objective:**

This randomized controlled trial examines the specific and combined effects of brief SS and TS interventions on older adults’ physical and mental health and pain intensity levels, among individuals living in nursing homes.

**Methods:**

A total of 96 patients aged 65-99 years from a nursing home were randomly assigned to 3 groups: SS, TS, and combined SS+TS interventions, each delivered as four 20-minute sessions. SS was implemented using a multisensory Snoezelen room. Pain intensity levels (per a Visual Analog Scale), blood pressure, heart rate, blood oxygen saturation, and hand grip strength (using a Jamar hand dynamometer) were measured before and after each of the 4 weekly therapeutic sessions. In addition, life satisfaction (per the Satisfaction with Life Scale) and anxiety (per the 7-item General Anxiety Disorder Scale) were evaluated before and after the whole intervention. Mixed model analyses tested the relative efficacy of the 3 interventions, applying simple slope analysis with Tukey correction. Study rationale and analytical plans were preregistered.

**Results:**

The combined intervention of SS and TS (SS+TS) resulted in reduced pain levels compared with SS (B=0.209, *P*=.006) and TS alone (B=0.23, *P*=.002) over 4 sessions (*F*_6,266_=2.62; *P*=.017; *R^2^*=0.23). Further, the combined SS+TS intervention resulted in reduced systolic blood pressure versus SS (B=0.09, *P*=.01) and TS alone (B=0.016, *P*<.001) groups (*F*_6,272_=5.42; *P*<.001; *R^2^*=0.29). In addition, the combined SS+TS intervention resulted in an increased grip strength versus SS (B=–0.35, *P*=.003) and TS alone (B=–0.032, *P*=.008) groups (*F*_6,273_=2.25; *P*=.04; *R^2^*=0.19). Moreover, combined SS+TS resulted in an improvement in life satisfaction (B=–4.29, *P*<.0001) compared with SS (B=–2.38, *P*=.0042) and TS alone (B=–1.20, *P*=.13) groups (*F*_2,39_=3.47; *P*=.04). Finally, SS+TS demonstrated greater improvement in symptoms of general anxiety disorder (B=10.64, *P*<.0001) compared with SS (B=3.30 *P*=.01) and TS alone (B=1.13, *P*=.37) (*F*_2,38_=13.5; *P*<.001) groups. No differences between the interventions were shown for blood oxygen saturation (*F*_6,273_=2.06; *P*=.06), diastolic blood pressure (*F*_6,272_=1.12; *P*=.35), and heart rate (*F*_6,273_=1.33; *P*=.23).

**Conclusions:**

The combined intervention of SS and TS showed therapeutic benefits for pain management and physical and mental health of older adults living in nursing homes, relative to each therapeutic component in isolation. This brief intervention can be readily implemented to improve well-being and optimize therapeutic resources in nursing home settings.

**Trial Registration:**

ClinicalTrials.gov NCT05394389; https://clinicaltrials.gov/ct2/show/NCT05394389

## Introduction

The global population is witnessing a notable rise in life expectancy, leading to an expanding aging population. According to the World Health Organization (WHO), older adults (aged 65 and above) made up 12.3% of the global population in 2017, with this proportion expected to surpass 22% by 2050 [1]. Similar trends are evident in the Middle Eastern region. For example, Israel’s older adult population (aged ≥65 years) accounted for approximately 12%-13% of its total population in 2020, mirroring trends observed in other developed countries. While the United Nations defines older persons as those aged 60 years and older, this study follows the widely accepted threshold of 65 years and older, commonly used in health-related studies and policies. This demographic shift has driven a significant increase in the number of nursing home beds globally, with the availability of hospital beds in nursing homes rising by more than 200% between 1988 and 2015 [2-5].

Individuals living in nursing homes often encounter challenges related to functional and cognitive impairments, stemming from the natural aging process, polypharmacy, and various health conditions [3,6]. Additionally, they may struggle with loneliness and emotional distress [7]. Therefore, a key therapeutic focus is on preserving and enhancing functional and cognitive abilities while promoting life satisfaction and overall quality of life [2].

Multisensory interventions, such as Snoezelen room therapy, alongside social support, are essential components of care for older adults in nursing homes [8]. Snoezelen room therapy is a form of multisensory stimulation (multi-SS) that involves the gradual application of sensory stimuli to enhance an individual’s sensory perception and integration. Numerous studies have emphasized the unique benefits of multi-SS interventions combined with therapist support (TS) in improving physical and mental health outcomes. These include positive effects on heart rate, blood pressure, blood oxygen saturation, depression, anxiety, and pain management [1,2,6,8].

In recent decades, there has been an increasing interest in using multisensory treatment approaches—incorporating visual, auditory, olfactory, and tactile stimulations—to improve the well-being of older adults. Originally developed in the 1960s and 1970s in the Netherlands and the United States for children with learning difficulties and developmental intellectual disabilities, multi-SS environments (MSSEs) have consistently shown benefits across diverse populations. Over time, the scope of MSSE has broadened to include older adult populations, demonstrating success in addressing conditions such as dementia and Alzheimer disease, as well as mental health concerns and other geriatric conditions [1,2,4,6].

In recent years, an expanding body of evidence has suggested that MSSE, especially through Snoezelen room therapy, shows promise as a valuable intervention for enhancing the well-being of older adults. MSSE interventions have been shown to improve communication with patients experiencing various communication disorders and a lack of expression, a common challenge among older adults living in nursing homes (eg, dementia) [1,7]. It creates a “listening” channel to internal sensations by enhancing individuals’ sensory regulation, which may serve as an alternative to verbal communication [8].

Previous research has consistently indicated that MSSE interventions, particularly Snoezelen room therapy, can lead to significant improvements in both physical and mental health. Physiological measures, such as heart rate and blood oxygen saturation, have shown positive changes after MSSE sessions [8-10]. Moreover, these interventions have been shown to be effective in enhancing psychosocial abilities and reducing agitation, behavioral disorders, anxiety, and depression among older adults. This, in turn, contributes to overall improvements in life satisfaction and fosters better social relationships and participation in meaningful activities [1,2,8,11].

**Figure 1:**
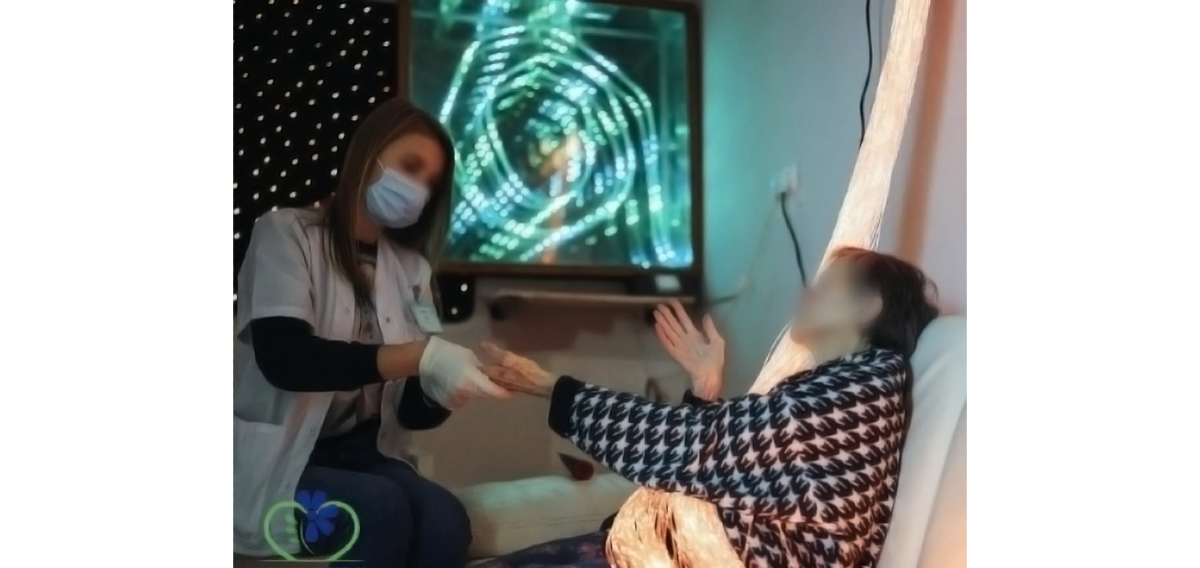
Demonstration of the Snoezelen room therapy.

An effective therapeutic tool within the MSSE approach is Snoezelen room therapy [8,11], which provides concentrated multi-SS while personalizing the intensity of the experience (Figure 1) [1,12,13].

The Snoezelen room is a specially designed space equipped with various sensory systems, providing a unique therapeutic environment for individuals. This innovative therapy involves the careful integration of lighting, sound, smell, and touch elements to create a multisensory experience. Guided by qualified therapists, individuals can participate in Snoezelen room sessions that evoke feelings of success, tranquility, pleasure, and contentment. One of the key strengths of the Snoezelen room intervention is its nonjudgmental and nonevaluative nature, offering a safe space where participants can explore their senses without fear of failure or criticism [4,14]. Beyond the previously mentioned benefits, Snoezelen room therapy has also been found to reduce pain sensations and enhance motivation, which is a crucial factor for participation in various daily activities [15].

Older adults may often experience social isolation and reduced social connections [16]. Social support has proven effective in alleviating depression, anxiety, and loneliness among older adults living in nursing homes, and it has also been shown to be a protective factor against depression [17,18]. Additionally, social support and social touch have a positive impact on reducing stress and pain intensity [19,20].

The role of therapists’ social support in enhancing the well-being of older adults, especially those in nursing homes, is increasingly recognized as a valuable intervention. Numerous studies have shown that the presence of dedicated and compassionate therapists can lead to significant improvements in self-esteem, pain perception, and overall quality of life among older adults [16,21]. A lack of social support among older individuals experiencing chronic pain can contribute to worsened mental health conditions and an increased risk of suicide [19,22]. In the context of nursing home care, there is a pressing need for targeted interventions that emphasize TS to effectively enhance the mental and physical well-being of older adults [16,17,23].

While both multi-SS and TS have been individually recognized for their potential to improve the well-being of older adults, the integration of these interventions remains relatively unexplored. Combining multi-SS with dedicated TS has the potential to create a more profound impact on the overall well-being of older individuals compared with each intervention used alone. By synergistically addressing both the sensory and social needs of older adults, this integrated approach may lead to better pain management and improved mental health outcomes.

In this study, we aim to address the existing research gap by exploring the potential benefits of an integrated intervention that combines multi-SS with TS for the care of older adults. This pilot randomized controlled trial (RCT) is designed to primarily assess the feasibility of implementing this combined intervention and to provide preliminary insights into its effectiveness. Our objectives are 2-fold: first, to determine whether this approach can be successfully implemented in nursing home settings, and second, to gather initial evidence on its potential for future large-scale trials. We will also evaluate the combined impact of SS and TS on pain management, as well as the overall physical and mental health of older adults. We hypothesize that the synergistic effect of integrating SS with TS will lead to more significant improvements in pain reduction and physical and mental health outcomes compared with either intervention applied in isolation.

## Methods

### Study Design

A randomized clinical trial was conducted over 1 year to examine the combined effect of SS and TS on pain intensity, as well as the mental and physical health of older adults living in nursing homes. The study specifically targeted older adults residing in these settings. All participants were randomly assigned into 3 groups by the supervising researcher using a stratification strategy to ensure balanced groups (see the “Randomization” section). The executing researcher and research staff responsible for assessments were blinded to the randomization process.

### Sampling Method and Randomization Process

Participants were recruited using a convenience sample from Ahuzat Hazafon Geriatric Nursing Hospital. Eligible participants were residents aged 65 years or older who had lived in the facility for at least three months and were capable of participating. Exclusion criteria included uncontrolled epilepsy and severe behavioral disturbances. Following eligibility screening, participants were assigned to 1 of 3 groups—SS, TS, or the combined SS+TS group—using stratified randomization. Stratification was based on age, sex, and cognitive level to ensure balanced groups. Randomization and allocation were overseen by an independent researcher who was not involved in the trial’s day-to-day activities. Additionally, research staff responsible for conducting baseline and postintervention assessments were blinded to group assignments to minimize assessment bias.

### Research Process

Patients, with the approval of their custodians, were invited to participate in the study. Each eligible participant attended 4 weekly therapeutic sessions based on their group assignment: SS (SS intervention using Snoezelen room therapy, involving gradual multi-SS without social interaction), TS (therapist’s social support intervention only, without SS), or SS+TS (a combined intervention of both SS and therapist’s social support). Each session lasted 20 minutes. Immediately before and after each session, we collected objective measures, including pain intensity, blood pressure, blood oxygen saturation, hand grip strength (HGS), and heart rate, from each participant. Additionally, eligible participants completed subjective assessments (Multimedia Appendix 1) to measure their satisfaction with life and anxiety levels at the beginning and end of the study.

### Sample Size

A prestudy power analysis was conducted, and the sample size was calculated based on an effect size of Cohen *d*=0.40, with a power of 0.80 and a type I error rate of 0.05/6 (0.0083) to account for 6 primary outcomes [18]. This analysis resulted in an estimated sample size of 102 participants.

### Outcome Measures

The objective measures included systolic and diastolic blood pressure, heart rate, blood oxygen saturation, and HGS. These metrics were assessed before and after each session. Patient-reported outcomes included life satisfaction, current pain, and general anxiety. Life satisfaction and general anxiety were assessed before the first session and after the last session, while pain was evaluated before and after each session.

### Research Tools

The following tools and instruments were used in this study:

- Blood pressure, heart rate, and blood oxygen saturation were measured using the “Welch Allyn” measuring instrument, a commonly used tool in clinical and research settings [24].
- HGS was assessed using the Jamar hand dynamometer, a valid and reliable tool for measuring muscle strength [25,26].
- Life satisfaction was measured using the Satisfaction with Life Scale (SWLS), a short 5-item instrument with high internal reliability (Cronbach α=0.93) and good test-retest reliability (0.84/0.80 for immediate/over a month interval) [27,28]. The SWLS has been translated into Hebrew and validated in multiple studies within the Israeli population, including older adults [29].
- Pain intensity was evaluated using the Visual Analog Scale, a 10-cm ruler with facial illustrations, which has been validated for pain assessment in older adults [30,31].
- General anxiety was assessed using the 7-item General Anxiety Disorder (GAD-7) Scale. The scale is considered valid and reliable for both clinical and research use, with a high level of internal reliability (Cronbach α=0.89). The literature demonstrates the effective use of the GAD-7 in the older population [32,33].
- The MMSE is widely used in Israel and has been validated in several studies involving older Israeli adults [34]. The Hebrew version of the MMSE demonstrated strong psychometric properties, including good internal consistency (Cronbach α=0.82) and excellent test-retest reliability across cognitive domains. It has been found to be a valid tool for assessing dementia in the Israeli older adult population [34].

### Prevention and Treatment of Selection Bias

To minimize selection bias, participants were recruited through a convenience sample, and stratified random assignment was used to evenly distribute potential confounders among the study groups. Additionally, the researcher conducting the study was blinded to the group assignments to reduce bias in data collection and analysis.

### Treatment of Confounders and Modifiers

To address confounding variables, statistical control was applied. Sex and age, known universal variables that can act as confounders, were included as control variables in the statistical analysis. This approach ensured that any observed group differences were not solely influenced by these variables.

### Data Analysis Methods

Group differences were examined using the chi-square test for categorical variables and a 1-way analysis of variance for continuous variables. For session-level outcomes, including blood pressure, blood oxygen saturation, heart rate, HGS, and pain intensity, percentage changes from presession to postsession were calculated. These changes were then analyzed using mixed models to test the differences between treatments over time, defining an interaction between time and treatment. Tukey-corrected post hoc analysis was applied for significant interactions. Pairwise contrasts were used to compare group differences at each session, with participant-based random intercepts and session slopes defined. The analysis of anxiety and life satisfaction outcomes was conducted using mixed effect models to compare pre- and postintervention differences across groups. When significant differences were detected, intervention-specific slopes were further examined using Tukey correction to account for multiple comparisons.

### Ethical Considerations and Approval

Participants were recruited by an occupational therapist who contacted all eligible patients living in the nursing home. The researcher explained the purpose of the study, ensured voluntary participation, and clarified that nonparticipation would not affect the patients’ or therapist-patient relationship. Informed consent was obtained from participants or their custodians, particularly for those lacking sufficient cognitive capacity. The study was preregistered with ClinicalTrials.gov (registered ID NCT05394389) and received ethical approval from the Faculty of Social Welfare & Health Sciences Ethics Committee at the University of Haifa (approval number [112/21]).

### Resources, Equipment, and Physical Tools

The study was conducted at Ahuzat Hazafon Geriatric Nursing Hospital, which provides medical, nursing, and paramedical services to its residents in mentally ill and nursing wards. The Snoezelen room where the study was conducted was a white, furnished space equipped with various SS systems, including lighting, audio, odor distribution, and massage systems. The room already had visual, auditory, tactile, and aromatic SS systems in place, which were used during the study.

## Results

### Participants

**Figure 2:**
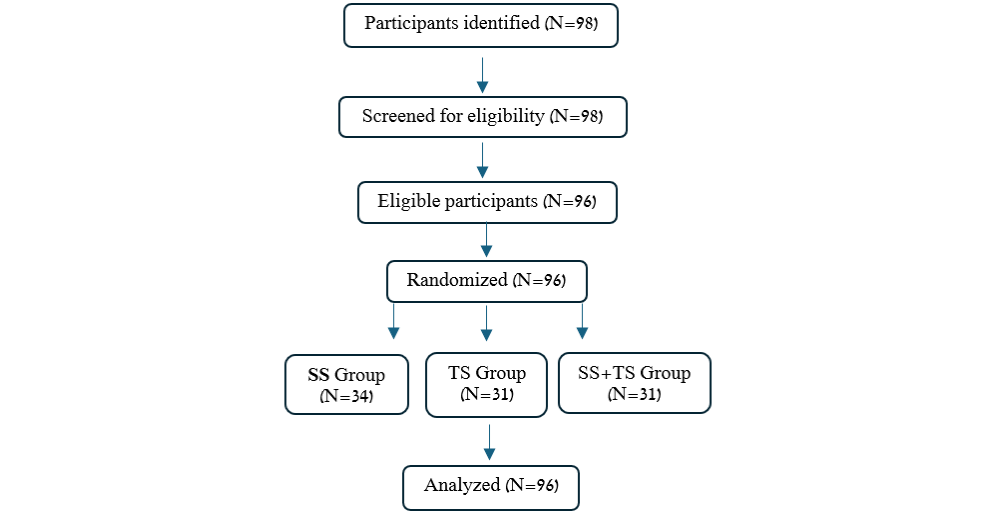
Flowchart of participants' recruitment.

The mean age of participants was 82.2 (SD 7.92) years, with the majority being women (62/96, 65%; Table 1). Most participants exhibited some degree of cognitive decline (mean Mini-Mental State Examination [MMSE] score 15.4, SD 8.45). The analysis indicated no significant differences between the groups in MMSE scores (*F*_2,93_=1.51; *P*=.32). Additionally, no significant differences were found among the 3 intervention groups for gender, age, cognitive state, or pain intensity (all *P*s>.2). Complete statistical analysis is presented in Multimedia Appendix 2. Participant recruitment is detailed in Figure 2.

**Table 1:** Sample characteristics of the study participants.

Characteristics		SS (n=34)	SS+TS (n=31)	TS (n=31)	Overall (N=96)
**Gender, n (%)**					
	Female	21 (62)	22 (71)	19 (61)	62 (65)
	Male	13 (38)	9 (29)	12 (39)	34 (35)
**Age (years)**					
	Mean (SD)	81.8 (8.34)	83.2 (7.47)	81.6 (8.03)	82.2 (7.92)
	Median (range)	84.5 (59.0-95.0)	84.0 (66.0-97.0)	83.0 (60.0-93.0)	84.0 (59.0-97.0)
**MMSE^a^= score**					
	Mean (SD)	13.8 (9.19)	15.5 (7.98)	17.0 (8.02)	15.4 (8.45)
	Median (range)	15.0 (0-27.0)	16.0 (0-27.0)	17.0 (0-29.0)	16.0 (0-29.0)
	Moderate pain, n (%)	10 (29)	8 (26)	9 (29)	27 (28)

^a^MMSE: Mini-Mental State Examination.

### Primary Outcomes

#### Comparing the Effect of SS, TS, and SS+TS Interventions on Physical Health

##### Blood Pressure

Intervention groups showed different temporal patterns of systolic blood pressure over the 4 sessions (*F*_6,272_=5.42; *P*<.001). The SS+TS group demonstrated a stronger reduction in systolic blood pressure compared with the SS alone group at session 3 (B=0.09; SE 0.03; *t*_272_=2.933; *P*=.01; Cohen *d*=0.97) and session 4 (B=0.073; SE 0.03; *t*_272_=2.4; *P*=.045; Cohen *d*=0.8). The SS+TS group also showed a stronger reduction in systolic blood pressure compared with the TS group at session 3 (B=0.107; SE 0.03; *t*_272_=1.71; *P*=.002; Cohen *d*=1.15) and session 4 (B=0.16; SE 0.031; *t*_272_=5.1; *P*<.001; Cohen *d*=1.70). All other group differences were not significant (all *P*>.2).

For diastolic blood pressure, the groups showed a similar pattern of improvement associated with the intervention (*F*_6,272_=1.12; *P*=.35).

##### Hand Grip

Intervention groups showed different temporal patterns of dominant HGS over the 4 sessions (*F*_6,273_=2.25; *P*=.04). In the post hoc analysis, the SS+TS group demonstrated a stronger improvement in dominant HGS compared with the SS alone group at session 3 (B=–0.35; SE 0.106; *t*_353_=–3.30; *P*=.003; Cohen *d*=0.89) and at session 4 (B=–0.34; SE 0.107; *t*_354_=–3.21; *P*=.004; Cohen *d*=0.87). The SS+TS group also showed stronger HGS improvement compared with TS at session 3 (B=–0.36; SE 0.107; *t*_353_=–3.87; *P*=.02; Cohen *d*=0.91) and at session 4 (B=–0.032; SE 0.107; *t*_354_=–2.99; *P*=.008; Cohen *d*=0.81). No significant differences were found in other comparisons between the groups (all *P*>.11). Nondominant HGS did not show group differences in temporal dynamics over the 4 sessions (*F*_6,271_=1.27; *P*=.27).

##### Pain

**Figure 3:**
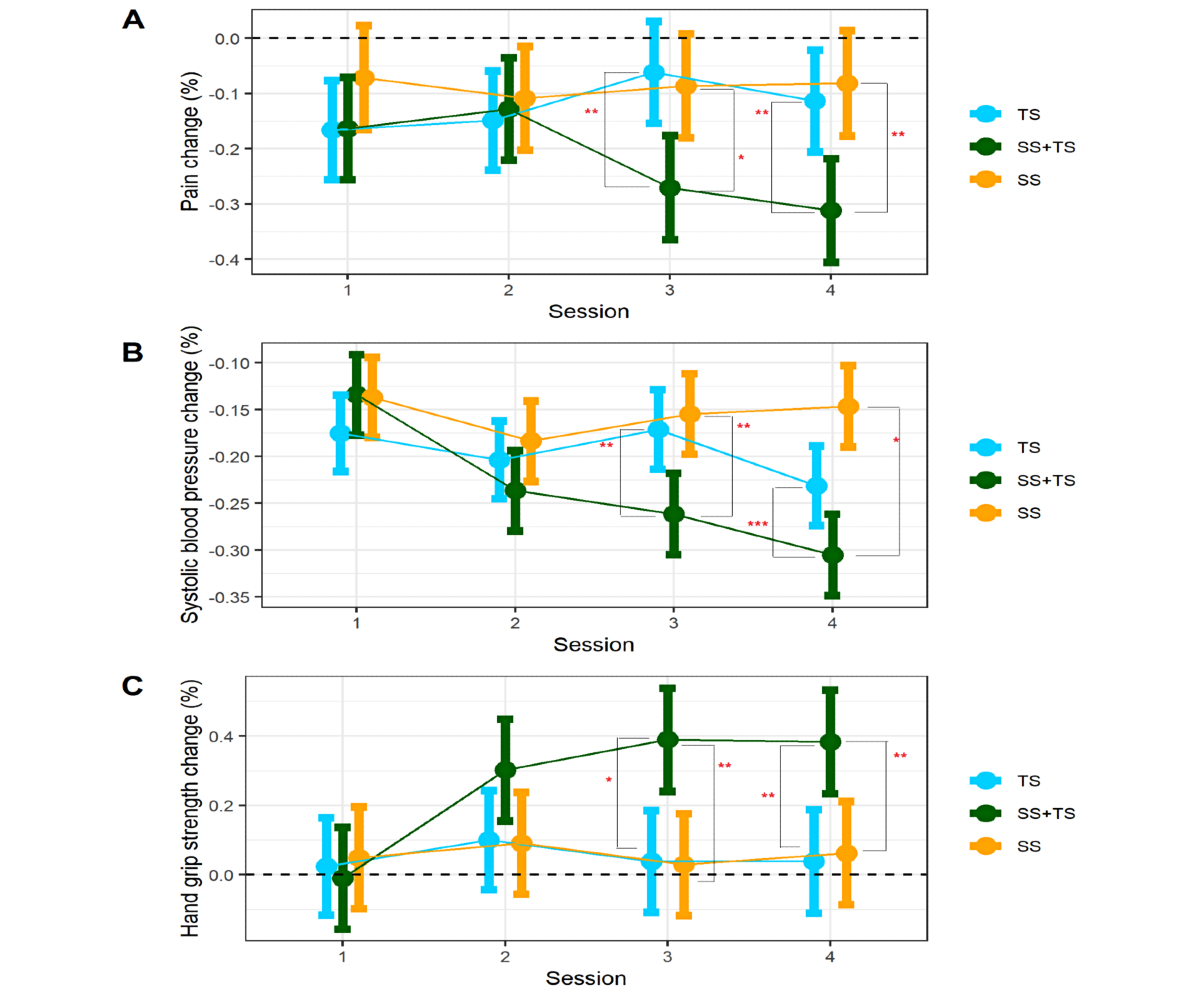
Fluctuations over 4 sessions for physical health measures: (A) pain intensity levels, (B) systolic blood pressure, and (C) hand grip across 3 interventions: (1) therapist support (TS), sensory stimulation (SS), sensory stimulation and therapist’s support (SS+TS). TS+SS demonstrated relative improvement in all 3 outcomes at sessions 3 and 4. *P<.05, **P<.01, ***P<.001.

Intervention groups showed different temporal patterns of pain reduction (*F*_6,266_=2.62; *P*=.02). The SS+TS group demonstrated stronger pain reduction compared with SS alone, at session 3 (B=0.209; SE 0.067; *t*_307_=3.12; *P*=.006; Cohen *d*=0.91) and at session 4 (B=0.20; SE 0.067; *t*_307_=2.96; *P*=.009; Cohen *d*=0.87). Additionally, the SS+TS group showed stronger pain reduction compared with the TS alone group, at session 3 (B=0.18; SE 0.068; *t*_307_=2.73; *P*=.02; Cohen *d*=0.81) and at session 4 (B=0.23; SE 0.068; *t*_307_=3.38; *P*=.002; Cohen *d*=1.01). No significant differences were found in all other pairwise group comparisons (*P*>.32 in all cases; Figure 3).

#### Comparing the Effect of SS, TS, and SS+TS Interventions on Emotional Health

##### Anxiety

The analysis revealed different temporal patterns of anxiety reduction among the intervention groups (*F*_2,38_=13.5; *P*<.001). As hypothesized, the SS+TS group demonstrated a significant reduction in anxiety (B=10.64; SE 1.41; *t*_38.8_=7.57; *P*<.001; Cohen *d*=1.57). By contrast, the SS and TS alone groups showed no significant improvement in anxiety (SS, *P*=.15; TS, *P*=.38).

##### Satisfaction With Life

**Figure 4:**
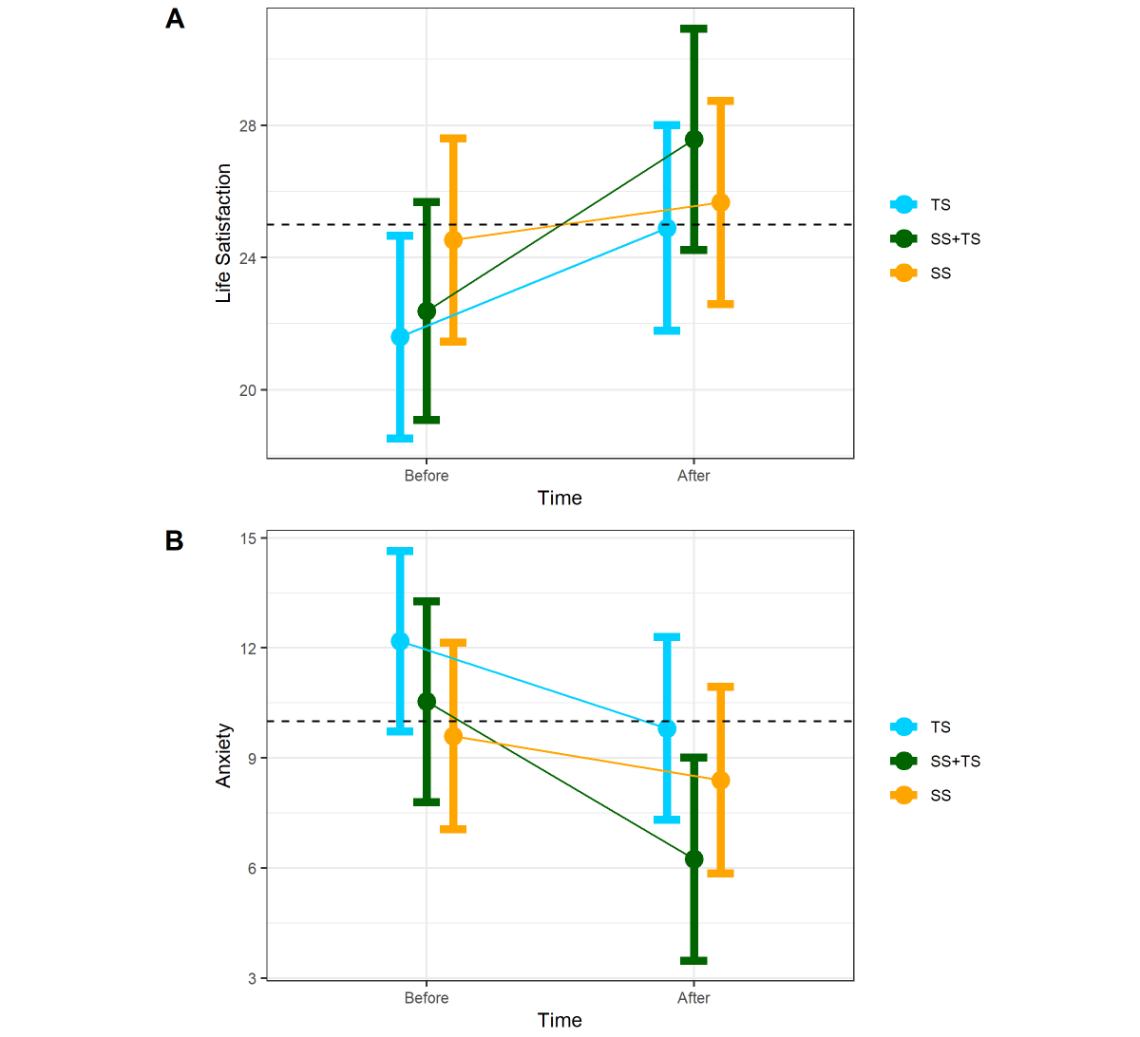
Mental health outcomes before/after 3 interventions: (1) therapist support (TS), (2) sensory Stimulation (SS), and (3) sensory stimulation and Therapist’s support (SS+TS). (A) Life satisfaction; (B) anxiety. The dotted line at life satisfaction=25 represents the cut-off for satisfied/extremely satisfied (above the line). The dotted line at anxiety=10 represents the cut-off for severe anxiety (above the line).

The analysis indicated different temporal patterns of satisfaction with life among the intervention groups (*F*_2,39_=3.47; *P*=.04). As hypothesized, the SS+TS group showed significant improvement in life satisfaction (SWLS; B=–4.29; SE 39.5; *t*_39.5_=–4.89; *P*<.001; Cohen *d*=1.44). Additionally, the SS group showed significant improvement in the SWLS (B=–2.38; SE 0.78; *P*=.004; Cohen *d*=0.99). However, the TS group did not show a significant improvement (*P*=.13; Figure 4).

### Secondary Outcomes

The analysis revealed that the groups showed a similar pattern of improvement related to the intervention for heart rate (*F*_6,271_=1.33; *P*=.23) and blood oxygen saturation (*F*_6,274_=2.06; *P*=.06).

## Discussion

### Principal Findings

This pilot randomized clinical trial is the first to investigate the combined effects of multi-SS in a Snoezelen room and TS (SS+TS) on the health of older adults living in nursing homes. Our findings indicate that the SS+TS intervention significantly reduced pain levels, lowered systolic blood pressure, and improved HGS compared with the SS and TS interventions individually. Additionally, the SS+TS intervention enhanced life satisfaction and reduced anxiety, with these effects being more pronounced than those observed with SS or TS alone [12,15,22].

Our findings supported the primary hypothesis, showing that the combined intervention of SS in a Snoezelen room, along with TS, had a more profound effect on pain intensity and physical and mental health outcomes compared with each intervention component delivered in isolation. Specifically, the SS+TS intervention led to reduced pain levels, lower systolic blood pressure, and increased HGS during sessions 3 and 4, surpassing the effects of SS and TS alone. Additionally, the SS+TS intervention showed greater improvements in life satisfaction and anxiety compared with the other interventions.

### Effect of Multisensory Stimulation and Therapist’s Support on Pain

The therapeutic impact of the combined SS+TS intervention notably improved pain experience during the latter half of the intervention. Previous research has highlighted the unique positive effects of SS and TS on pain perception [1,15]. Pharmacological interventions, particularly opioids and other pain medications, can have adverse effects on older adults, including falls, cognitive impairment, constipation, and addiction [23,35]. Nonpharmacological methods for pain management, such as the Snoezelen room integrated with enhanced TS, offer a safer alternative and are often more cost-effective in the long term compared with continuous pharmaceutical treatments [23,33,34].

Our findings, combined with existing literature on the Snoezelen room intervention, support the multidimensional effects of the SS+TS intervention on pain intensity. Alleviating pain is crucial, as the experience of pain can lead to decreased mobility, sleep disturbances, emotional distress, and, consequently, a reduced quality of life among older adults [23,35,36].

Interestingly, our results revealed that the therapeutic effects of the combined intervention were most pronounced during sessions 3 and 4. This observation aligns with prior research indicating that pain reduction following nonpharmacological interventions can be delayed [1,15,37-39]. For example, pain reduction resulting from physical exercise for low back pain is often gradual and occurs over time [40,41].

Additionally, social support–focused interventions have been associated with delayed pain relief [23,37]. This delay may be due to the gradual implementation and refinement of coping strategies. It can take time for participants, particularly older adults, to adjust to a new type of therapy that includes multiple elements. This adaptation period may delay the positive interpretation of bodily sensations [37,42-46]. Further investigation is warranted to explore the temporal dynamics and long-term effects of the intervention.

### Effect of Multisensory Stimulation and Therapist’s Support on Physical Health

We observed an increased impact of the combined SS+TS intervention on systolic blood pressure and HGS. Previous studies have shown that SS, especially olfactory and visual stimulation, can effectively reduce blood pressure and enhance overall health [12,47,48]. Another study highlighted that TS can enhance physical health by providing a safe and nonjudgmental space for older adults to share their concerns, fears, and anxieties. Such support can help reduce stress, which is linked to various physical health issues, including hypertension, heart disease, and compromised immune function [17,23,38,49-52].

Our findings underscore the importance of interventions that address the mind-body connection. Recent studies have shed light on the physiological mechanisms through which mental processes impact the immune system and other bodily functions [47,53-55]. Moreover, some multisensory interventions include resistance training and muscle-strengthening exercises. Participating in these activities can help older adults maintain or even improve muscle strength and endurance, leading to better physical health and greater independence in daily activities [54,56].

Contrary to our initial hypothesis, we did not find that the combined SS+TS intervention provided greater benefits compared with SS and TS alone in terms of heart rate, blood oxygen saturation, and diastolic blood pressure. None of the interventions showed improvements in these metrics. It is plausible that the duration of our intervention (4 sessions lasting 17-20 minutes each) was insufficient to detect changes in these outcomes. Moreover, existing research supports the notion that similar interventions focusing on sensory processing and social support may not effectively improve heart rate and blood oxygen saturation in advanced age [8,21]. Additionally, stable diastolic blood pressure among older adults is often indicative of good health, and reducing diastolic blood pressure could even pose a risk factor for heart disease [57,58].

### Improving Mental Health Through Multisensory Stimulation and Therapist Support

Our study yielded promising findings regarding the therapeutic benefits of a brief SS+TS intervention for enhancing mental health among older individuals residing in nursing homes. This intervention showed potential for improving life satisfaction and reducing anxiety, without exacerbating the common issue of polypharmacy in the geriatric field. It is well-established that better mental health in this population is closely linked to improvements in physical well-being [49]. Previous research has consistently highlighted that multi-SS, which often includes elements such as soothing music, aromatherapy, and tactile sensations, can reduce stress levels, alleviate anxiety, and promote a sense of calm. For individuals dealing with anxiety disorders, such as generalized anxiety disorder or posttraumatic stress disorder, multisensory interventions can serve as valuable coping mechanisms.

Multisensory experiences can trigger the release of endorphins and other feel-good neurotransmitters, leading to improved mood and emotional well-being. Furthermore, chronic pain has been associated with an increased aversiveness to unpleasant multi-SSs, suggesting shared mechanisms between pain intensity and multisensory processing [15,59]. For older individuals who may struggle with depression or mood disorders, regular participation in multisensory activities can offer a natural, nonpharmacological approach to improving their mood. Additionally, multisensory activities that encourage social engagement can help mitigate feelings of isolation and loneliness, providing residents with a sense of belonging and companionship [19,21,32,60]. Moreover, these improvements have been shown to contribute to an enhanced sense of well-being and increased social engagement [60]. An extensive body of research has demonstrated the significant role of TS in improving various markers of mental health [17,18,20,61]. TS is widely regarded as a valuable component for addressing emotional and social needs, which are often critical for older adults facing mental health challenges. Emotional support and empathy can significantly enhance treatment outcomes for older adults experiencing depression, anxiety, and reduced life satisfaction [18,20,62,63].

An intriguing question arises from our study: What makes the combined SS+TS intervention particularly effective? We propose that the therapeutic state of mind fostered by the relaxing environment created through SS plays a crucial role. This environment prepares the groundwork for the TS to have a greater impact by reducing patient resistance. Supporting this notion, previous research has demonstrated that SS creates a more conducive analgesic environment compared with standard settings [15,64]. This shift in environment may be particularly therapeutic for older individuals living in nursing homes, where the atmosphere often mirrors that of a hospital, characterized by medications and medical procedures that can heighten feelings of discomfort and anxiety.

The observed effects of the SS+TS intervention may result from an interactive synergy between SS and TS. Rather than being merely additive, these interventions appear to enhance each other, leading to greater therapeutic outcomes. The soothing environment created by SS likely amplifies the effectiveness of TS, enabling a more profound impact on participants’ health. This combined effect underscores the potential of the SS+TS intervention to be particularly effective in settings such as nursing homes, where establishing a calming, supportive environment is crucial.

In exploring the nature of the combined effects of SS and TS, a critical question arises: Do these interventions provide additive or interactive benefits when applied together? An additive effect would suggest that the benefits of the combined SS+TS intervention are simply the sum of the individual impacts of SS and TS when applied separately. By contrast, an interactive effect would indicate a synergistic relationship, where the combined SS+TS intervention produces outcomes that surpass the sum of its individual components. Although a no-treatment control group would offer a more definitive basis for distinguishing between these possibilities, our current analysis, based on a visual examination of pre-to-post intervention changes, suggests the presence of an interactive effect. This observation suggests that the combination of SS and TS may activate mechanisms that go beyond their individual contributions, leading to enhanced therapeutic outcomes. This synergy could stem from how SS prepares individuals mentally and physically for more effective engagement with therapeutic support, or vice versa. Future research with a more comprehensive experimental design, including a control group and separate groups for each intervention, would be invaluable for confirming and further elucidating these effects.

### Limitations and Recommendations for Future Studies

Although our study has provided valuable insights, it is important to acknowledge its limitations. Conducted in a single nursing home, our findings may not fully represent the broader population.

First, the use of a convenience sample from a single nursing home may introduce selection bias, as participants may not fully represent the broader population of older adults in similar care settings. For instance, participants with behavioral issues were excluded, which limits the generalizability of the findings to this wider population. Additionally, the voluntary nature of participation may introduce selection bias, as participants who chose to join the study may have been more motivated or in better health, potentially influencing the outcomes. Future studies should aim to replicate our findings across multiple settings, explore additional measures of physical and mental health, and extend the intervention period to assess long-term effects.

Second, the sustainability of the observed effects was not assessed, as follow-up data were not collected. Consequently, while our findings suggest potential benefits of the combined SS+TS intervention, the duration of these effects remains unknown.

Third, the study’s modest sample size may limit the power to detect small effect sizes. Further research with larger sample sizes and extended follow-up periods is needed to validate these preliminary findings. Furthermore, exploring the impact of the SS+TS intervention on medication use, particularly for mental health conditions, as well as on sleep quality among nursing home residents, would provide additional valuable insights into the intervention’s effectiveness.

### Conclusion and Implications for Clinical Practice

Our study highlights the therapeutic potential of the combined SS+TS intervention for managing pain and enhancing the physical and mental well-being of older adults in nursing homes. The results indicate that this intervention not only improves key health outcomes but also has the potential to reduce reliance on medications, addressing concerns related to polypharmacy. The scalability and ease of implementation make SS+TS a valuable nonpharmacological option for nursing homes, potentially enhancing patient care and resource efficiency. Future research should focus on larger-scale studies and long-term follow-up to confirm these findings and explore the broader applicability of the intervention.

## Appendix

Multimedia Appendix 1 Research protocols.

Multimedia Appendix 2 Additional analysis and results.

Multimedia Appendix 3 CONSORT 2010 (Consolidated Standards of Reporting Trials) checklist.

## Abbreviations

GAD-7: 7-item General Anxiety Disorder
HGS: hand grip strength
MMSE: Mini-Mental State Examination
MSSE: multisensory stimulation environment
SS: sensory stimulation
SWLS: Satisfaction with Life Scale
TS: therapist support
WHO: World Health Organization
