# Supplementary material for: The Combined Effect of Multisensory Stimulation and Therapist Support on Physical and Mental Health of Older Adults Living in Nursing Homes: Pilot Randomized Controlled Trial
Source: J Med Internet Res. 2025 Jan 14;27:e55042. doi: 10.2196/55042 (PMC11775494; doi:10.2196/55042)
Supplement: Multimedia Appendix 1 [file jmir_v27i1e55042_app1.docx]

**1.1. Protocol for sensory-stimulating therapy in the Snoezelen room:**

1. Each patient will go through an intervention that consists of 4 sessions, each session lasting 17-20 minutes. The period between the meeting is a week.

2. Before the session, a test of the outcome measures such as blood pressure, grip strength, stochasticity, heart rate, life satisfaction (SWLS), VAS, GAD7 and wellbeing index should be performed on specific devices and in a uniform method (detailed in the methodology section).The metrics based on the patient's report will be passed first, then we will pass objective metrics, so that the patient's reporting is not affected by the knowing of blood pressure, heart rate, saturation and grip strength indices.

3. The sessions will take place at Ahuzat Hazafon Geriatric Nursing Hospital, Occupational Therapy Department - in the Snoezelen Room.

4. The patient will be transferred to the Snoezelen room by his occupational instructor.

5. An explanation will be given to the patient about the treatment he is going to undergo, and his consent will be obtained before the intervention begins.

*The explanation is: Dear patient, you have now arrived at the Snoezelen room, I am going to turn on some functions that give sensory stimulation to the various sensory systems (sight, hearing, smell). In order, to soak up a warm, relaxing atmosphere and encourage general sensory regulation and encourage a sense of well-being.*

*I am going to turn off the white light and activate these functions gradually, the duration of the intervention is a total of 17 -20 minutes, for any question or requests I am here, wishing you a pleasant experience*

6. The patient will enter the Snoezelen room when the room is lit by normal lighting only and for safety reasons, two minutes should be allowed to look at the room and the items in it.

7. Sensory stimulation will be given gradually in this order: visual sensory stimulation, then add audio stimulation and then add aromatic stimulation, approval must be obtained from the patient at any stage for which stimulation is pleasant and does not overwhelm him.

8. Uniform visual, aromatic, and auditory sensory stimulation will be used for all patients, and Uniform volume for all patients as well.

9. A "Please do not disturb" sign must be hung on the door of the room, and the patient must be supervised while in the room to maintain his safety.

10. Bringing food and drinks into the room is not allowed.

11. Bringing telephones and electronic devices into the room is not allowed.

12. Treatment should be submitted only individually.

13. A soap bubble device with interchangeable lighting, a professional light replacement device hanging on a wall, and a light replacement device in professional pastel colors will be used on the patient as a visual sensory stimulation.

14. A professional gentle fragrance blend will be used, as an aromatic sensory stimulation by dripping into an odor diffuser in a regulated manner.

15. Relaxing music from the natural world will be used and the music will be played on a professional speaker from Sony, placed in the Snoezelen room.

16. In this intervention group the patient will stay in the room under supervision only without any human intervention and any Initiating interaction (apart from the technical intervention of operating the various devices and providing an answer to the various requests such as stopping the session).

17. Sensory stimulation should be stopped gradually as well, in the following order: first inform the patient that the session is over, turn down the background music, turn the off-soap bubble fixture, turn on natural lighting, turn the off light fixture in alternating pastel colors and gradually reduce odor intensity.

18. The Snoezelen room must be kept clean and tidy after use and the follow-up form must be signed, specifying the date, time, name, and signature.

19. The patient should be transferred back to his or her housing unit by the same occupational instructor who brought him or her.

20. After the session, a test of the outcome measures such as blood pressure, grip strength, stochasticity, heart rate, life satisfaction (SWLS), VAS, GAD7 and wellbeing index should be performed on specific devices and in a uniform method (detailed in the methodology section).The metrics based on the patient's report will be passed first, then we will pass objective metrics, so that the patient's reporting is not affected by the knowing of blood pressure, heart rate, saturation and grip strength indices.

**1.2. Protocol for sensory-stimulating therapy with social interaction in Snoezelen**

**room:**

1. Each patient will go through an intervention that consists of 4 sessions, each session lasting 17-20 minutes. The period between the meeting is a week.

2. Before the session, a test of the outcome measures such as blood pressure, grip strength, stochasticity, heart rate, life satisfaction (SWLS), VAS, GAD7 and wellbeing index should be performed on specific devices and in a uniform method (detailed in the methodology section).The metrics based on the patient's report will be passed first, then we will pass objective metrics, so that the patient's reporting is not affected by the knowing of blood pressure, heart rate, saturation and grip strength indices.

3. The sessions will take place at Ahuzat Hazafon Geriatric Nursing Hospital, Occupational Therapy Department - in the Snoezelen Room.

4. The patient will be transferred to the Snoezelen room by his occupational instructor.

5. An explanation will be given to the patient about the treatment he is going to undergo, and his consent will be obtained before the intervention begins.

*The explanation is: Dear patient, you have now arrived at the Snoezelen room, I am going to turn on some functions that give sensory stimulation to the various sensory systems (sight, hearing, smell). In order to soak up a warm, relaxing atmosphere and encourage general sensory regulation and encourage a sense of well-being. I am going to turn off the white light and activate these functions gradually, the duration of the intervention is a total of 17-20 minutes, for any question or request I am here, wishing you a pleasant experience*

6. The patient will enter the Snoezelen room when the room is lit by normal lighting only and for safety reasons, two minutes should be allowed to look at the room and the items in it. An explanation will be given for the process that the patient is going to go through by his or her occupational instructor.

7. Sensory stimulation will be given gradually in this order: visual sensory stimulation, then add audio stimulation, then add aromatic stimulation and then tactile stimulation, approval must be obtained from the patient at any stage for which stimulation is pleasant and does not overwhelm him.

8. Uniform visual, aromatic, auditory, and tactile sensory stimulation will be used for all patients, and Uniform volume for all patients as well.

9. In this research group, the occupational instructor will make sure to have a conversation with the patient about his or her life, feelings, encouraging a pleasant memory of the past, while providing touch and tactile sensory stimulation by a gentle massage to the hands.

*Guiding questions when conducting the conservation:*

*Concentrate on talking about the patient, especially around his feelings, raising memories from the past and bringing them to the present, by asking questions such as:*

*- Tell me how you feel?*

*- Are you enjoying yourself right now?*

*- Tell me about pleasant memories from the past ...*

*- Remember happy events and share with me if you want…*

10. A dialogue will be conducted with the patient in a low tone, at the pace that the patient dictates and to the extent of the development that the patient wants. Of course, with extra care to maintain the patient's dignity and to entrust good listening and positive reinforcements.

11. The care provider must pay attention! Do not correct the patient if he observed forgetting, confused, saying unrelated to a situation, and let him express himself freely, according to the principles of the validation approach.

12. A "Please do not disturb" sign must be hung on the door of the room, and the patient must be supervised while in the room to maintain his safety.

13. Bringing food and drinks into the room is not allowed.

14. Bringing telephones and electronic devices into the room is not allowed.

15. Treatment should be submitted only individually.

16. A soap bubble device with interchangeable lighting, a professional light replacement device hanging on a wall, and a light replacement device in professional pastel colors will be used on the patient as a visual sensory stimulus.

17. A professional gentle fragrance blend will be used, as an aromatic sensory stimulation by dripping into an odor diffuser in a regulated manner.

18. Relaxing music from the natural world will be used and the music will be played on a professional speaker from Sony, placed in the Snoezelen room.

19. Gentle tactile sensory stimulation will be applied by a gentle massage to the hands using a medical board cream for the hands.

20. Sensory stimulation should be stopped gradually as well, in the following order: first, inform the patient that the session is over, stop tactile stimulation, turn down background music, turn off soap bubble fixture, turn on natural lighting, turn off light fixture in alternating pastel colors and gradually reduce odor intensity.

21. The Snoezelen room must be kept clean and tidy after use and the follow- up form must be signed, specifying the date, time, name, and signature

22. The patient should be transferred back to his or her housing unit by the same occupational instructor who brought him or her, after a summary of the meeting and sharing of the patient's feelings after the session.

23. After the session, a test of the outcome measures such as blood pressure, grip strength, stochasticity, heart rate, life satisfaction (SWLS), VAS, GAD7 and wellbeing index should be performed on specific devices and in a uniform method (detailed in the methodology section). The metrics based on the patient's report will be passed first, then we will pass objective metrics so that the patient's reporting is not affected by the knowledge of blood pressure, heart rate, blood oxygen saturation, and grip strength indices.

**1.3. Protocol for social support\interaction only:**

1. Each patient will go through an intervention that consists of 4 sessions, each session lasting 17-20 minutes. The period between the meeting is a week.

2. Before the session, a test of the outcome measures such as blood pressure, grip strength, stochasticity, heart rate, life satisfaction (SWLS), VAS, GAD7, and wellbeing index should be performed on specific devices and in a uniform method (detailed in the methodology section). The metrics based on the patient's report will be passed first, then we will pass objective metrics so that the patient's reporting is not affected by the knowledge of blood pressure, heart rate, ,saturation and grip strength indices.

3. The sessions will take place at Ahuzat Hazafon Geriatric Nursing Hospital, Occupational Therapy Department - in the Snoezelen Room.

4. The patient will be transferred to the Snoezelen room by his occupational instructor.

5. An explanation will be given to the patient about the treatment he is going to undergo, and his consent will be obtained before the intervention begins.

*The explanation is Dear patient, you have now arrived at the Snoezelen room, I am going to conduct a conversation with you To soak up a warm, relaxing atmosphere and encourage a sense of well-being. the duration of the intervention is a total of 17-20 minutes, for any questions or request I am here, wishing you a pleasant experience*

6. The patient will enter the Snoezelen room when the room is lit by normal lighting only! the whole session will be held when only white light is lit (the entire room is painted white as well as the furniture), without the use of the various light fixtures. An explanation will be given about the process that the patient is going to go through by his or her occupational instructor.

7. In this research group, the occupational instructor will make sure to have a conversation ^with^ the patient about his or her life, feelings, encouraging a pleasant memory of the past, while providing tactile sensory stimulation by a gentle massage to the hands.

*Guiding questions when conducting the conservation:*

*Concentrate on talking about the patient, especially around his feelings, raising memories from the past and bringing them to the present, by asking questions such as:*

*- Tell me how you feel?*

*- Are you enjoying yourself right now?*

*- Tell me about pleasant memories from the past ...*

*-Remember happy events and share with me if you want…*

8. A dialogue will be conducted with the patient in a low tone, at the pace that the patient dictates and to the extent of the development that the patient wants. Of course, with extra care to maintain the patient's dignity and to entrust good listening and positive reinforcements.

9. The care provider must pay attention! Do not correct the patient if he observed forgetting, confused, or saying unrelated to a situation, and let him express himself freely, according to the principles of the validation approach.

10. A "Please do not disturb" sign must be hung on the door of the room, and the patient must be supervised while in the room to maintain his safety.

11. Bringing food and drinks into the room is not allowed.

12. Bringing telephones and electronic devices into the room is not allowed.

13. Treatment should be submitted only individually.

14. The patient should be transferred back to his or her housing unit by the same occupational instructor who brought him or her, after a summary of the meeting and sharing of the patient's feelings after the session.

15. The Snoezelen room must be kept clean and tidy after use and the follow-up form must be signed, specifying the date, time, name, and signature.

16. After the session, a test of the outcome measures such as blood pressure, grip strength, stochasticity, heart rate, life satisfaction (SWLS), VAS, GAD7, and well-being index should be performed on specific devices and in a uniform method (detailed in the methodology section). The metrics based on the patient's report will be passed first, then we will pass objective metrics so that the patient's reporting is not affected by the knowing of blood pressure, heart rate, blood oxygen saturation, and grip strength indices.
