# Supplementary material for: The Combined Effect of Multisensory Stimulation and Therapist Support on Physical and Mental Health of Older Adults Living in Nursing Homes: Pilot Randomized Controlled Trial
Source: J Med Internet Res. 2025 Jan 14;27:e55042. doi: 10.2196/55042 (PMC11775494; doi:10.2196/55042)
Supplement: Multimedia Appendix 2 [file jmir_v27i1e55042_app2.docx]

**2.1. Hand grip**

**Dominant hand:**

           Sum Sq  Mean Sq NumDF   DenDF F value Pr(>F)

__________________________________________________________________________

SESSION_c       1.3677 0.45589     3 273.625  2.9031 0.0352838 *

GROUP           2.6463 1.32313     2  94.142  8.4257 0.0004303 ***

SESSION_c:GROUP 2.1167 0.35279     6 273.634  2.2465 0.0391858 *

**__________________________________________________________________________**

**Non-dominant hand**

|  |
| --- |

| Sum Sq  Mean Sq NumDF   DenDF F value Pr(>F)    SESSION   0.37594 0.125315 3 271.316 1.8746 0.13413  GROUP           0.39040 0.195202 2  94.301   2.9200 0.05883 .  SESSION_c:GROUP 0.50979 0.084966   6 271.320  1.2710 0.27084    ^ Type III Analysis of Variance Table with Satterthwaite's method  **Post hoc dominant:** |
| --- |
|  |
| **session findings** |
| Estimate (B) SE  df t.ratio p.value Cohen's D |

**1**

contrasts

1-2 0.03304 0.103 352   0.320  0.9452

1-3 -0.02584 0.103 352  -0.250  0.9661

2-3 -0.05887 0.106 352  -0.557  0.8428

**2**

contrasts

1-2 -0.20217 0.104 352  -1.943  0.1281

1-3 0.00893 0.104 352   0.086  0.9959

2-3 0.21110 0.106 352   1.998  0.1141

**3**

contrasts

1-2 -0.35146 0.106 353  -3.300  0.0030

1-3 0.00914 0.106 353   0.087  0.9959

2-3 0.36061 0.107 353   3.386  0.0023

**4**

contrasts

1-2 -0.34474 0.107 354  -3.211  0.0041

1-3 -0.02395 0.107 354  -0.223  0.9729

2-3 0.32079 0.107 353   2.988  0.0084

^Degrees-of-freedom method: kenward-roger

P value adjustment: tukey method for comparing a family of 3 estimates

**2.2. Pain:**

Type III Analysis of Variance Table with Satterthwaite's method:

            Sum Sq  Mean Sq NumDF   DenDF F value Pr(>F)

__________________________________________________________________________

SESSION_c       0.08819 0.029397    3 266.496  0.5643 0.63900

GROUP           0.49866 0.249331     2  90.035  4.7861 0.01058 *

SESSION_c:GROUP 0.81995 0.136659     6 266.507 2.6233 0.01732 *

**Post – hoc pain:**

|  |
| --- |
| **session findings** |

| Estimate (B) SE  df t.ratio p.value Cohen's D |
| --- |

**1**

contrasts

1-2     -0.00278 0.0654 307   -0.042  0.9990 -0.0122

1-3 -0.09506 0.0660 307  -1.441  0.3214 -0.4165

2-3 -0.09229 0.0670 305  -1.376  0.3545 -0.4043

**___________________________________________________________________**

**2**

contrasts

1-2 -0.02066 0.0655 307  -0.316  0.9466 -0.0905

1-3 -0.03997 0.0660 307  -0.605  0.8173 -0.1751

2-3 -0.01930 0.0670 305   -0.288   0.9553 -0.0846

**___________________________________________________________________**

**3**

contrasts

1-2 0.20860 0.0669 312   3.119  0.0056 0.9139

1-3 0.02456 0.0669 310   0.367  0.9285 0.1076

2-3 -0.18404 0.0675 307   -2.725  0.0186 -0.8063

**___________________________________________________________________**

**4**

contrasts

1-2 0.19826 0.0669 312   2.964  0.0091 0.8686

1-3 -0.03199 0.0675 312  -0.474   0.8835 -0.1401

2-3 -0.23025 0.0681 309   -3.383  0.0023 -1.0088

___________________________________________________________________

Degrees-of-freedom method: kenward-roger

P value adjustment: tukey method for comparing a family of 3 estimates

**2.3. blood pressure:**

**systolic blood pressure:**

Type III Analysis of Variance Table with Satterthwaite's method:

           Sum Sq  Mean Sq NumDF   DenDF F value Pr(>F)

__________________________________________________________________________

SESSION_c         0.31582 0.105274   3 272.102 12.1121 1.825e-07 ***

GROUP     0.10256 0.051278   2  93.895  5.8997  0.003859 **

SESSION_c:GROUP 0.28258 0.047097   6 272.109 5.4186 2.609e-05 ***

**Post – hoc systolic blood pressure:**

|  |
| --- |
| **session findings** |

| Estimate (B) SE  df t.ratio p.value Cohen's D |
| --- |

**1**

contrasts

1-2 -0.04103 0.0301 243  -1.364  0.3614 -0.4401

1-3 -0.03838 0.0301 243  -1.276  0.4101 -0.4117

2-3 0.00265 0.0308 243   0.086  0.9959 0.0284

**___________________________________________________________________**

**2**

contrasts

1-2 0.03275 0.0303 245   1.082  0.5260 0.3513

1-3 -0.02014 0.0303 245  -0.665  0.7838 -0.2160

2-3 -0.05288 0.0308 243  -1.719  0.2002 -0.5672

**___________________________________________________________________**

**3**

contrasts

1-2 0.09038 0.0308 253   2.933  0.0102 0.9695

1-3 -0.01652 0.0306 251  -0.539  0.8519 -0.1772

2-3 -0.10690 0.0310 246  -3.453  0.0019 -1.1467

**___________________________________________________________________**

**4**

contrasts

1-2 0.07393 0.0308 253   2.399  0.0451 0.7930

1-3 -0.08478 0.0308 254  -2.752  0.0174 -0.9094

2-3 -0.15871 0.0311 249  -5.096  <.0001 -1.7024

___________________________________________________________________

**diastolic blood pressure:**

Type III Analysis of Variance Table with Satterthwaite's method:

           Sum Sq  Mean Sq NumDF   DenDF F value Pr(>F)

__________________________________________________________________________

SESSION_c    0.109481 0.036494 3 272.70 2.1308 0.0966

GROUP     0.013813 0.006907     2  93.55  0.4033 0.6693

SESSION_c:GROUP 0.115063 0.019177     6 272.71  1.1197 0.3509

**2.4. blood oxygen saturation:**

Type III Analysis of Variance Table with Satterthwaite's method:

SumSq  Mean Sq NumDF   DenDF F value Pr(>F)

__________________________________________________________________________

SESSION_c    5.2268  1.7423     3 274.692   1.9435 0.12282

GROUP   3.8097   1.9048     2  93.789  2.1249 0.12516

SESSION_c:GROUP 11.0834   1.8472     6 274.703  2.0606 0.05805 .

**2.5. heart rate:**

Type III Analysis of Variance Table with Satterthwaite's method:

SumSq  Mean Sq NumDF   DenDF F value Pr(>F)

__________________________________________________________________________

SESSION_c   0.012269 0.0040898   3 271.434  0.7449 0.5261

GROUP   0.002916 0.0014578     2  92.808  0.2655 0.7674

SESSION_c:GROUP 0.043821 0.0073036     6 271.442  1.3303 0.2437

**2.6. Satisfaction with life scale – SWLS:**

Type III Analysis of Variance Table with Satterthwaite's method:

SumSq  Mean Sq NumDF   DenDF F value Pr(>F)

__________________________________________________________________________

SESSION_c   143.693 143.693     1 39.048 31.0005 2.055e-06 ***

GROUP   10.256   5.128     2 48.924  1.1063   0.33891

SESSION_c:GROUP 32.207  16.104     2 39.039  3.4742   0.04087 *

**Post – hoc SWLS:**

|  |
| --- |
| **GROUP findings** |
| var.trend    SE   df t.ratio p.value Cohen's D |

 1         -2.38 0.784 39.5  -3.035  0.0042 0.99

 2         -4.29 0.876 39.5  -4.899  <.0001 1.44

3     -1.20 0.786 39.0   -1.526   0.1350 0.35

**2.7. Anxiety –GAD:**

Type III Analysis of Variance Table with Satterthwaite's method:

SumSq  Mean Sq NumDF   DenDF F value Pr(>F)

__________________________________________________________________________

SESSION_c   517.92  517.92     1 38.191 43.1778 9.201e-08 ***

GROUP   24.53   12.27     2 55.392  1.0225  0.3664

SESSION_c:GROUP 324.06   162.03     2 38.176 13.5081 3.663e-05 ***

Signif. codes:  0 ‘***’ 0.001 ‘**’ 0.01 ‘*’ 0.05 ‘.’ 0.1 ‘ ’ 1

> test(emtrends(GAD_s, ~GROUP, var = "var") )

**Post – hoc GAD:**

|  |
| --- |
| **GROUP findings** |
| var.trend    SE   df t.ratio p.value Cohen's D |

 1         3.30 1.30 38.8   2.535  0.0154 0.97

 2         10.64 1.41 39.0   7.571   <.0001 1.57

 3        1.13 1.26 38.1   0.896  0.3758 0.49

**Table A: Pain**

| **Session number** | **Group** | **Marginal means** | **SE** |
| --- | --- | --- | --- |
| 1 | SS | -16.62 | 4.55 |
| 1 | SS+TS | -16.34 | 4.70 |
| 1 | TS | -7.11 | 4.78 |
| 2 | SS | -14.85 | 4.55 |
| 2 | SS+TS | -12.79 | 4.70 |
| 2 | TS | -10.86 | 4.78 |
| 3 | SS | -6.15 | 4.69 |
| 3 | SS+TS | -27.01 | 4.77 |
| 3 | TS | -8.61 | 4.78 |
| 4 | SS | -11.32 | 4.69 |
| 4 | SS+TS | -31.15 | 4.77 |
| 4 | TS | -8.12 | 4.85 |

**Table B: Systolic Blood Pressure**

| **SESSION number** | **GROUP** | **Marginal means** | **SE** |
| --- | --- | --- | --- |
| 1 | SS | -17.5 | 2.08 |
| 1 | SS+TS | -13.4 | 2.18 |
| 1 | TS | -13.7 | 2.18 |
| 2 | SS | -20.4 | 2.10 |
| 2 | SS+TS | -23.6 | 2.18 |
| 2 | TS | -18.4 | 2.18 |
| 3 | SS+TS | -26.1 | 2.20 |
| 3 | TS | -15.5 | 2.18 |
| 4 | SS | -23.1 | 2.16 |
| 4 | SS+TS | -30.5 | 2.20 |
| 4 | TS | -14.6 | 2.20 |

**Table C: Diastolic Blood Pressure**

| **SESSION number** | **GROUP** | **Marginal means** | **SE** |
| --- | --- | --- | --- |
| 1 | SS | -0.04 | 0.02 |
| 1 | SS+TS | 0.02 | 0.02 |
| 1 | TS | -0.007 | 0.02 |
| 2 | SS | -0.06 | 0.02 |
| 2 | SS+TS | -0.04 | 0.02 |
| 2 | TS | -0.04 | 0.02 |
| 3 | SS | -0.01 | 0.02 |
| 3 | SS+TS | -0.06 | 0.02 |
| 3 | TS | -0.03 | 0.02 |
| 4 | SS | -0.08 | 0.02 |
| 4 | SS+TS | -0.04 | 0.02 |
| 4 | TS | -0.03 | 0.02 |

**Table D: Grip Strength (Right Hand)**

| **SESSION number** | **GROUP** | **Marginal means** | **SE** |
| --- | --- | --- | --- |
| 1 | SS | 2.35 | 7.13 |
| 1 | SS+TS | -0.94 | 7.47 |
| 1 | TS | 4.94 | 7.47 |
| 2 | SS | 10.02 | 7.24 |
| 2 | SS+TS | 30.23 | 7.47 |
| 2 | TS | 9.12 | 7.47 |
| 3 | SS | 3.79 | 7.47 |
| 3 | SS+TS | 38.94 | 7.59 |
| 3 | TS | 2.88 | 7.47 |
| 4 | SS | 3.87 | 7.59 |
| 4 | SS+TS | 38.35 | 7.59 |
| 4 | TS | 6.27 | 7.59 |

**Table E: Grip Strength (Left Hand)**

| **SESSION number** | **GROUP** | **Marginal means** | **SE** |
| --- | --- | --- | --- |
| 1 | SS | -0.04 | 0.05 |
| 1 | SS+TS | -0.10 | 0.05 |
| 1 | TS | -0.009 | 0.05 |
| 2 | SS | 0.12 | 0.05 |
| 2 | SS+TS | -0.03 | 0.05 |
| 2 | TS | -0.01 | 0.05 |
| 3 | SS | 0.001 | 0.05 |
| 3 | SS+TS | -0.10 | 0.05 |
| 3 | TS | 0.03 | 0.05 |
| 4 | SS | 0.12 | 0.05 |
| 4 | SS+TS | -0.08 | 0.05 |
| 4 | TS | -0.02 | 0.05 |

**Table F: Saturation**

| **SESSION number** | **GROUP** | **Marginal means** | **SE** |
| --- | --- | --- | --- |
| 1 | SS | -0.006 | 0.16 |
| 1 | SS+TS | -0.01 | 0.17 |
| 1 | TS | -0.005 | 0.17 |
| 2 | SS | -0.007 | 0.16 |
| 2 | SS+TS | -0.01 | 0.17 |
| 2 | TS | 0.002 | 0.17 |
| 3 | SS | 0.007 | 0.17 |
| 3 | SS+TS | -0.01 | 0.17 |
| 3 | TS | -0.002 | 0.17 |
| 4 | SS | 0.83 | 0.17 |
| 4 | SS+TS | -0.01 | 0.17 |
| 4 | TS | -0.01 | 0.17 |

**Table G: SWLS**

| **GROUP** |  | **Marginal means** | **SE** |
| --- | --- | --- | --- |
| SS | before | 21.600 | 1.527800 |
| SS+TS | before | 22.385 | 1.641120 |
| TS | before | 24.533 | 1.527800 |
| SS | after | 24.896 | 1.550220 |
| SS+TS | after | 27.583 | 1.669183 |
| TS | after | 25.667 | 1.527800 |

**Table H: GAD**

| **GROUP** |  | **Marginals means** | **SE** |
| --- | --- | --- | --- |
| SS | before | 12.19 | 1.23 |
| SS+TS | before | 10.54 | 1.36 |
| TS | before | 9.60 | 1.26 |
| SS | after | 9.80 | 1.24 |
| SS+TS | after | 6.24 | 1.38 |
| TS | after | 8.40 | 1.27 |
